# Comparative analyses of monocyte memory dynamics from mice to humans

**DOI:** 10.1007/s00011-023-01762-8

**Published:** 2023-07-15

**Authors:** Ziyue Yi, Shuo Geng, Liwu Li

**Affiliations:** Department of Biological Sciences, Virginia Tech, 149 Life Science 1 Bldg, Blacksburg, VA 24061-0910 USA

**Keywords:** Innate immune memory, Monocyte subsets, Comparative single cell analyses, Chronic and acute inflammatory diseases, Leukocyte therapeutics, Monocyte memory dynamics, Comparative analyses, Exhaustion, Sepsis, Trajectory

## Abstract

**Background:**

Innate monocytes can adopt dynamic “memory” states ranging from low-grade inflammation to pathogenic exhaustion, dependent upon signal strength and history of challenges. Low-grade inflammatory monocytes facilitate the pathogenesis of chronic inflammatory diseases, while exhausted monocytes drive the pathogenesis of severe sepsis. Although clinical and basic studies suggest the conservation of key features of exhausted monocytes from human and murine sepsis, systems analyses of monocyte exhaustion among human and murine monocytes are lacking.

**Methods:**

We performed cross examination of septic monocytes scRNAseq data recently collected from human sepsis patients as well as experimental septic mice, in reference to monocytes experimentally exhausted in vitro. Furthermore, we performed pseudo-time analyses of in vitro programmed monocytes following prolonged challenges causing either low-grade inflammation or exhaustion. Additional comparative analyses of low-grade inflammatory monocytes were performed with scRNAseq data from selected human patients with chronic low-grade inflammatory diseases.

**Results:**

Our systems analyses reveal key features of monocyte exhaustion including reduced differentiation, pathogenic inflammation and immune suppression that are highly conserved in human and murine septic monocytes, and captured by in vitro experimental exhaustion. Pseudo-time analyses reveal that monocytes initially transition into a less-differentiated state with proliferative potential. The expansion of proliferative monocytes can be observed not only in experimentally challenged monocytes, but also in tissues of murine sepsis and human septic blood. We observed that monocytes similarly transition into the less-differentiated state when challenged with a subclinical dose endotoxin under chronic inflammatory conditions. Instead of being exhausted, monocytes with prolonged challenges with super-low dose endotoxin bifurcate into the low-grade inflammatory immune-enhancing or the chemotactic/adhesive state, often see in atherosclerosis or auto-immune diseases.

**Conclusions:**

Key features of monocyte memory dynamics are identified and conserved in human and murine monocytes, which can be captured by prolonged challenges of innate signals with varying signal strength.

## Introduction

Emerging studies reveal the presence of innate immune memory, a complex process during which innate leukocytes adopt altered activation states depending upon the signal strength and history of challenges [1]. Basic and translational studies suggest that monocyte memory dynamics occur in both human and murine monocytes, with shared features of altered activation dynamics ranging from priming, tolerance to exhaustions. However, comparative analyses of monocyte memory dynamics have not been systematically conducted.

In the context of signal strength and history-dependent memory of lipopolysaccharide (LPS), our group defined that persistent challenges of monocytes with super-low dose LPS drive the sequential activation of cell proliferation, inflammatory activation and adhesion [2, 3], which correspond to the pathogenesis of chronic inflammatory diseases, such as atherosclerosis [2]. In contrast, prolonged challenges with higher doses of LPS promote monocyte proliferation and immune exhaustion, as reflected in the tripartite features of reduced differentiation; immune suppression; and pathogenic inflammation [4]. Pathologically, less-mature exhausted monocytes fail to differentiate into functional macrophages with effective clearance of pathogens and cellular debris. Exhausted monocytes have reduced expression of immune-enhancing mediators, such as CD86, CD80, and MHCII, while highly potent in expressing immune-suppressive mediators such as PD-L1 that collectively compromise immune defense against pathogens. Adding further damage to the host, exhausted monocytes express pathogenic inflammatory mediators including various cytokines as well as CD38, an NAD depleting enzyme [4]. These key exhausted features have been increasingly observed in experimental sepsis as well as human sepsis patients [5–7]. Despite their conserved natures, currently there is no available comparative analysis to cross-examine monocyte exhaustion among murine and human systems.

To fill this critical void, we aim to perform comparative analyses of key exhaustion features of monocytes from murine and human sepsis, in comparison with the in vitro experimental model of monocyte exhaustion. Based on recent scRNAseq data from human sepsis [8–11] and murine model of cecal ligation and puncture sepsis [12], we cross-examined key gene expression profiles representing the tripartite characteristics of monocyte exhaustion identified in the in vitro model of exhaustion. To determine potential mechanisms, we performed pseudo-time analyses of monocyte exhaustion based on our existing scRNAseq analyses of exhausted monocytes, mapping out the trajectory of monocyte memory dynamics dependent on signal strength. We identified key representative genes involved in proliferation, pathogenic inflammation and immune suppression similarly modulated in experimentally exhausted monocyte in vitro, as well as experimental sepsis and human septic patients. To complement the analyses of low-grade inflammatory monocytes challenged with chronic weak signals associated with chronic inflammatory diseases, such as lupus, we cross-examined key low-grade inflammatory gene signatures from scRNAseq data collected from human lupus patients. Collectively, our systems analyses reveal highly conserved natures of monocyte exhaustion across murine and human septic monocytes in vivo and in vitro, as well as shared features of low-grade inflammation from monocytes collected from auto-immune disease patients.

## Materials and methods

### Experimental data

The experimental data used in this study were obtained either from publicly available databases or previously published in our own laboratory. Murine data were described in our previous publication [4] and deposited under the accession number GSE190856. Human data were obtained from recently published sources [8–11], including the Single Cell Portal SCP548, Array Express E-MTAB-9357, GSE148020, and GSE198616. Murine monocytes harvested from septic heart were as reported [12].

### Data analysis

The data obtained were preprocessed and analyzed using the Seurat package as we described previously [4]. Briefly, the raw data were normalized using the ‘LogNormalize’ function, and variable genes were identified using the ‘FindVariableFeatures’ function with the default parameters. Principal component analysis (PCA) was performed using the variable genes, and the top 10–20 principal components were used for clustering analysis. The ‘FindClusters’ function was used to cluster the cells based on their gene expression profiles with a resolution around 0.1. The clusters were visualized using Uniform Manifold Approximation and Projection (UMAP) and default clustering by Seurat.

Differential Expression Genes (DEG) analysis was conducted under Seurat default setting. Dot plot analyses were conducted to examine represented genes defining fundamental characteristics of distinct memory monocyte subsets.

Dot plots displaying the relative expression levels of selected targets were generated with the Seurat software through the unbiased default setting, representing both the percentages of target gene expression among the cell sub-populations as well as normalized expression levels of the target gene as we previously described [4]. Key selected target genes differentially expressed in a target cluster were obtained for each cluster using the non-parametric Wilcoxon rank sum test in R. The dot size represents the percentage of cell population expressing the target gene and the dot color intensity represents the normalized expression level of the given target.scVelo (v0.2.5) analyses were performed on murine scRNAseq data previously deposited and published [4], which were then aligned by Cellranger (v3.1.0), reevaluated by Velocyto (v0.17), and then sent to scVelo to predict the vectors.

GO analyses were conducted using the Enrichment Gene Ontology database to identify the most enriched and appropriate GO terms for the DEG analysis results as we previously described [2].

## Results

### Three cardinal features of monocyte exhaustion recapitulated in vitro through prolonged stimulation with high dose bacterial endotoxin

Clinical studies with human septic patients and animal studies of sepsis increasingly suggest the expansion of less-matured monocytes with pathogenic inflammatory and immune-suppressive characteristics [13]. We recently published the generation of exhausted monocytes through in vitro culture of murine bone marrow monocytes with persistent challenges of higher dose endotoxin [4]. However, key genes involved in the exhaustion and underlying mechanisms are not thoroughly understood. To further systematically characterize the exhausted monocytes, we focused on classes of genes selectively involved in modulating cell differentiation/proliferation, pathogenic inflammation, and immune suppression, three pillars of monocyte exhaustion in sepsis.

**Fig. 1:**
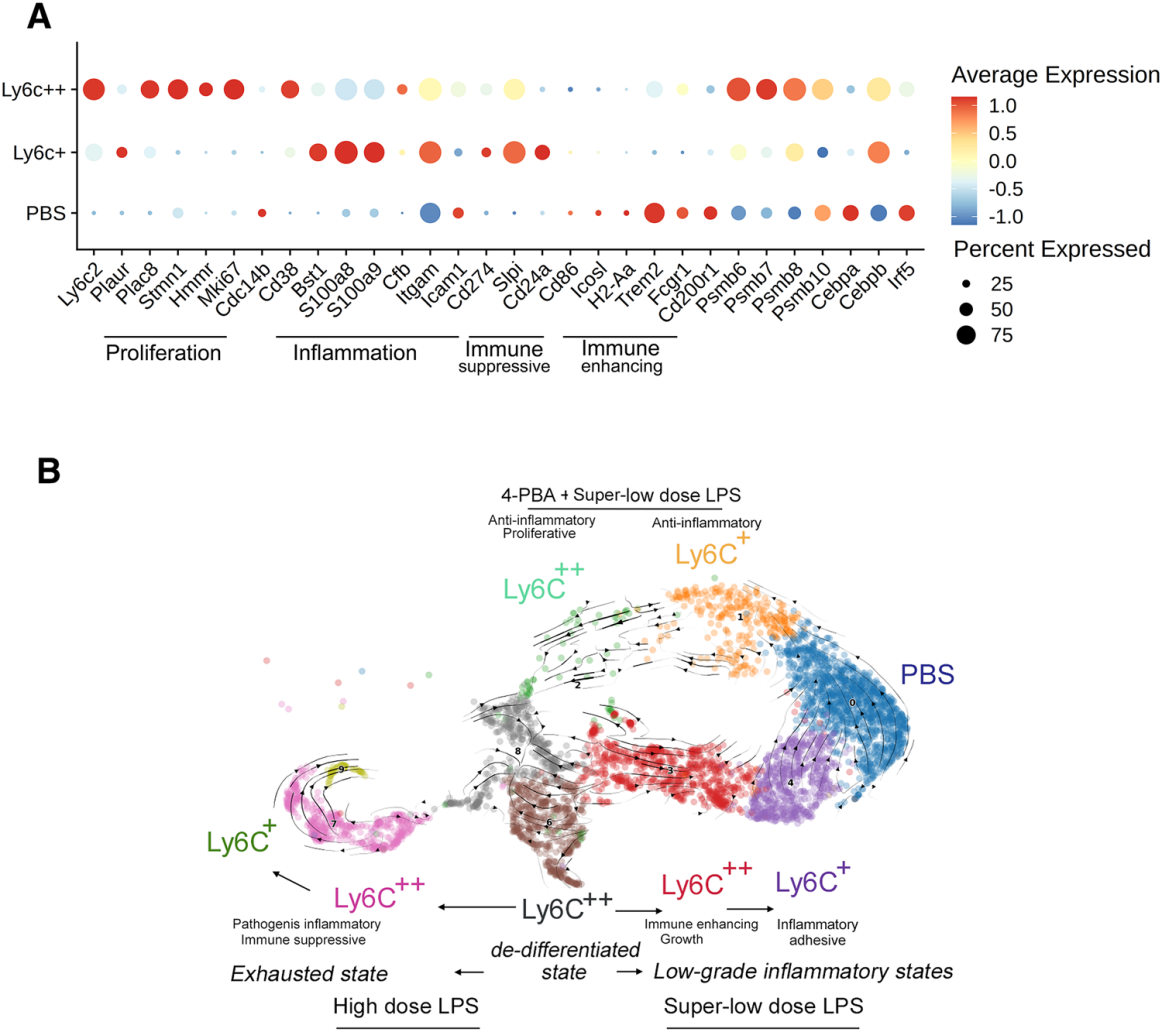
Analyses of monocyte memory dynamics in vitro. **A** scRNAseq data collected from in vitro exhausted monocytes with 5-day high dose LPS treatment were re-analyzed to capture key representative genes involved in proliferation, pathogenic inflammation, immune suppression or immune enhancing, as well as selected regulatory genes. **B** scRNAseq data collected from monocytes treated with 5-day challenges of high dose LPS, super-low dose LPS, or 4-PBA plus super-low dose LPS were clustered together and used for the scVelo analyses, revealing their dynamics pseudo-time trajectory

As shown in Fig. 1A, in vitro exhausted monocytes (both of the Ly6C^+^ and Ly6C^++^ groups) potently express higher levels of genes involved in proliferation (elevated *Plac8*, *Plaur*, *Stmn1*, *Hmmr*, *Mki67* and reduced *Cdc14b*), pathogenic inflammation (*Cd38*, *Bst1*, *Itgam, Icam1*), and immune suppression (elevated *Cd274, Slpi, CD24a* and reduced *Cd86*, *Icosl and H2-Aa*). Among genes involved in cell proliferation, STMN1 interacts with microtubules and is an indicator of mitosis, while MKI67 condenses in the nucleus during mitosis. PLAC8 and PLAUR are also known to be involved in cell proliferation [14–16]. On the other hand, CDC14B was recently shown to promote cell differentiation and prevent excessive proliferation [17]. Among immune suppressive genes, CD274 (PD-L1) protein potently suppresses T cell functions [18], SLPI and CD24A proteins are involved in anti-inflammatory immune suppression [19–21]. In contrast, CD86, ICOSL and H2-Aa proteins are involved in enhancing adaptive immune functions [22–24]. Among pathogenic inflammatory genes, both CD38 and BST1 proteins lead to the depletion of cellular metabolic fuel NAD and the generation of inflammatory secondary mediators [25, 26]. CD11B and ICAM1 proteins are involved in inflammatory adhesion of monocytes to vasculatures [27, 28]. Furthermore, as less-differentiated monocytes, in vitro exhausted monocytes lose phagocytic receptors *Trem2*, *Fcgr1* and immune modulator *Cd200r1*. Collectively, in vitro exhausted monocytes exhibit altered gene expression profiles of reduced differentiation, enhanced pathogenic inflammation and immune suppression.

To gain further mechanistic insights of their generation, we looked at key signaling components and transcription factors modulating monocyte behaviors. Proteasomes have been known to be elevated in activated monocytes [29]. We observed elevation of proteasome components such as Psmb6 and Psmb10 in exhausted monocytes. SOCS3 protein is known to be elevated in “tolerant” monocytes with reduced expression of immune-enhancing mediators [30], and we observed elevated Socs3 in exhausted monocytes. In terms of transcription factors, C/EBPα and C/EBPβ proteins are known to be differentially involved in monocyte differentiation and activation, with C/EBPα facilitating cell differentiation [31, 32] and C/EBPβ promoting inflammatory activation [33]. C/EBPα is also involved in suppressing the generation of myeloid derived suppressor cells [34]. We observed that *C/ebpα* is reduced in exhausted monocytes, correlated with reduced differentiation. In contrast, *C/ebpβ* is elevated in exhausted monocytes, corresponding to elevated pathogenic inflammation. IRF5 protein is a key transcription factor promoting the expression of immune-enhancing genes, such as *Cd86, H2-Aa* and *Cd40* [35–37]. Exhausted monocytes exhibit levels of *Irf5*, corresponding to reduced expression of *Cd86* and *H2-Aa*.

One of the advantages in analyzing single cell data is the potential to perform pseudo-time analyses for their ontogeny and trajectory. We then performed scVelo analyses of monocytes with prolonged challenges of either super-low dose or high dose LPS, or an anti-inflammatory agent 4-PBA together with super-low dose LPS (Fig. 1B). Our analyses reveal that cells initially prompted into the proliferative, less differentiated state following either super-low or high dose LPS. Subsequently, monocytes track into the exhausted Ly6C^++^ monocytes and gradually into the Ly6C^+^ exhausted monocytes with lower proliferating potential.

In contrast, monocytes challenged with prolonged signals of super-low dose LPS bifurcate into a totally different direction. Our analyses reveal that low-dose LPS treated monocytes first move into the Ly6C^++^ monocytes with growth-promoting and immune-enhancing state, and then into the Ly6C^+^ chemotactic and adhesive low-grade inflammatory state [2]. In our previous experimental studies, we applied anti-inflammatory mediator 4-PBA and reported that 4-PBA blocked the generation of inflammatory monocytes induced by super-low dose LPS. 4-PBA has also been independently shown to potently reduce atherosclerosis [7, 38, 39], and its mechanisms of action require future clarification. Although 4-PBA may have diverse functions, the usage of 4-PBA in this particular system of monocyte dynamics can robustly arrest monocyte activation. In the lens of monocyte dynamics, monocyte arrested by 4-PBA provides a novel reference point for examining monocyte activation dynamics, in guiding future studies of monocyte single cell trajectories. We superimposed 4-PBA plus low-dose LPS scRNAseq onto the scVelo map, and further validated that 4-PBA arrests low-dose LPS treated monocytes into the proliferating or anti-inflammatory state.

### Septic human monocytes share similar gene expression profiles of monocyte exhaustion

**Fig. 2:**
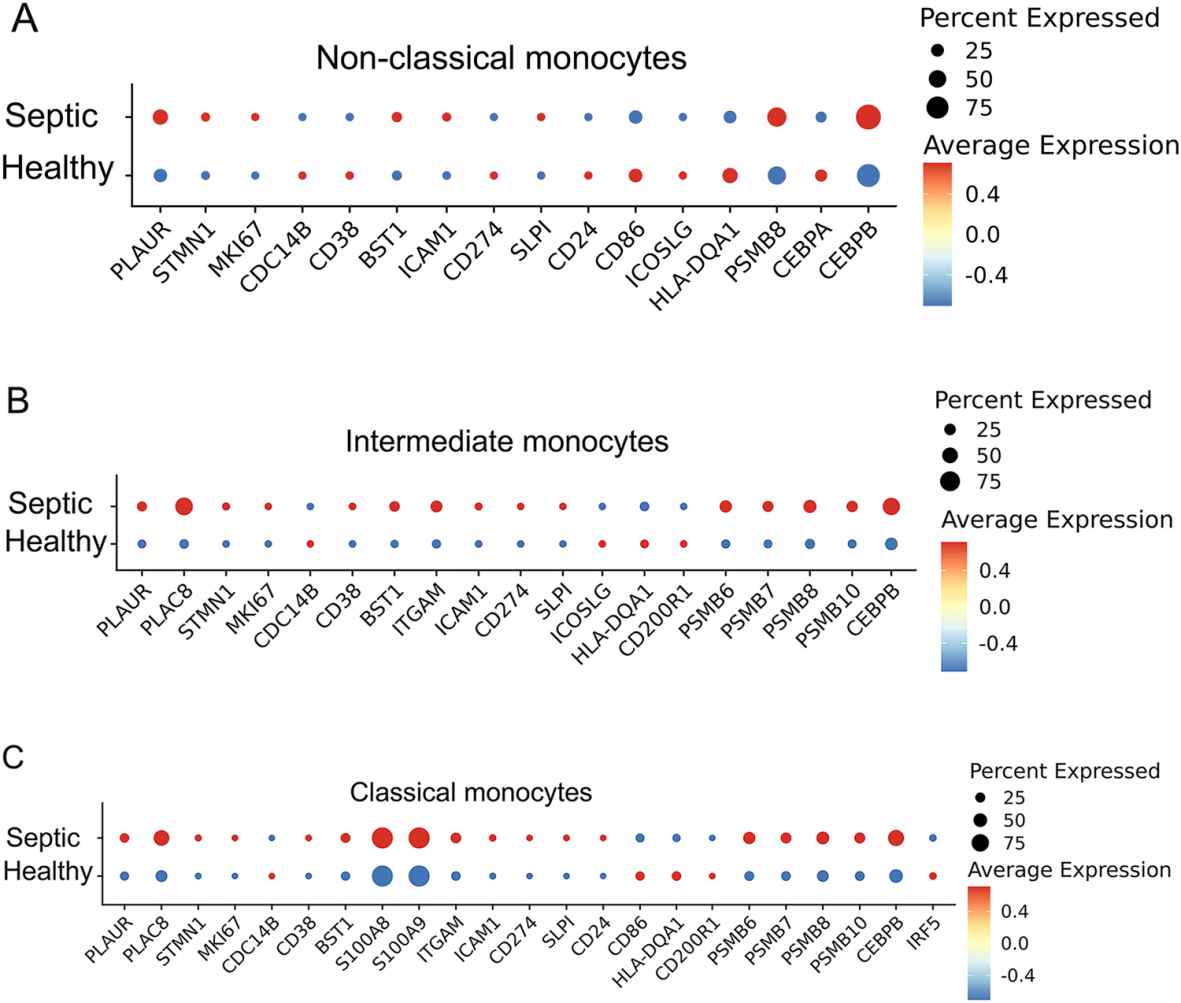
Capturing of key exhaustion maker genes from each subset of monocytes from septic patients. **A** Dot plot analyses capturing selected genes comparing the non-classical subset of monocytes from healthy or septic patient blood. **B** Dot plot analyses capturing selected genes comparing the intermediate subset of monocytes from healthy or septic patient blood. **C** Dot plot analyses capturing selected genes comparing the classical subset of monocytes from healthy or septic patient blood

With recently available scRNAseq data of human septic monocytes [9], we then performed comparative analyses of key genes representing monocyte exhaustion among murine and human systems. Human monocytes can be separated into classical, intermediate, and non-classical subpopulations by evaluating the levels of the CD14 and FCGR3A (CD16) markers. We analyzed the relative expression of above-described genes identified in exhausted murine monocytes among human monocyte subsets from healthy and septic patients [9] (Fig. 2).

As reported independently, septic patients exhibit a reduction of the non-classical monocytes, and an expansion of the intermediate and classical monocytes [9, 40]. We then further separately compared gene expression profiles based on the scRNAseq clusters of each monocyte subset. As shown in Fig. 2, the less-differentiated signatures (elevated *STMN1*, *MKI67* and reduced *CDC14B*) can be seen to be elevated in septic non-classical monocytes, suggesting the conserved characteristic of reduced differentiation and proliferative potential of septic monocytes. The immune-enhancing genes such as *CD86*, *ICOSL* and *HLA-DQA1* are all reduced suggesting the development of immune suppression. Key transcription factor *C/EBPα* involved in differentiation as well as the transcription factor *IRF5* involved in immune-enhancing gene expression were both reduced in septic non-classical monocytes. Among the expanded intermediate and the classical subsets, septic monocytes also exhibit elevated levels of proliferative genes (*STMN1*, *MKI67*). Furthermore, additional pathogenic inflammatory genes are elevated which include *CD38, BST1*, *ICAM1, ITGAM*, as well as the immune suppression gene *CD274* (PD-L1). Mechanistically, proteasome components such as *PSMB6* and *10* as well as the pathogenic inflammatory transcription factor *C/EBPβ* were all elevated in the intermediate and classical subsets of septic monocytes as compared to healthy subsets (Fig. 2). Together, our targeted analyses confirm that human septic monocytes shared the cardinal features of experimentally exhausted monocytes with reduced differentiation; pathogenic inflammation and immune suppression, key attributes leading to compromised host defense and multi-organ injuries.

### Cardinal features of monocyte exhaustion identified are also conserved in the experimental model of animal sepsis

During the preparation of this manuscript, an independent study appeared in *Nature Metabolisms* that provided single cell profiling of infiltrating macrophages and monocytes in murine septic heart tissues [12]. The authors focused on interpreting the alterations of tissue macrophages in a time-dependent fashion following the initial insult of cecal ligation and puncture, with an initial depletion of TREM2 positive macrophages and rapid restoration of TREM2 macrophages 7–21 days post the initial sepsis. Although the authors did not elaborate, we noticed that there was a sustained expansion of infiltrating monocytes within the septic heart lasting throughout the 21-day observation period and never restore back to the baseline. There is also an expansion of a highly proliferative “cycling cell” population that is also sustained throughout the 21-day post-sepsis period.

**Fig. 3:**
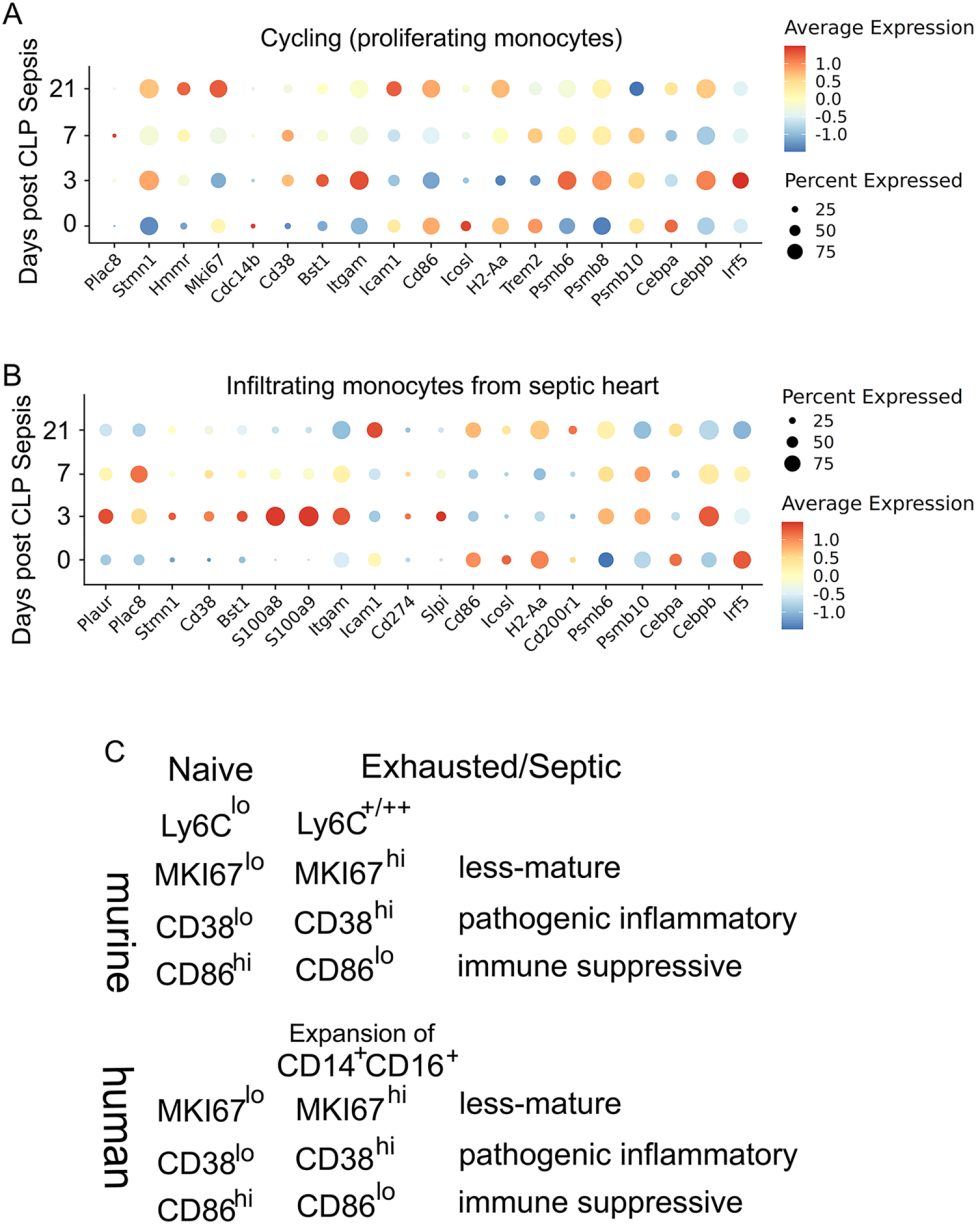
Analyses of monocyte memory dynamics collected from septic animals. scRNAseq data collected from mice subjected to cecal ligation and puncture were used for focused analyses of either cycling myeloid cells (**A**) or infiltrating monocytes (**B**) in the heart tissue. Representative genes were captured in the dot plot. **C** Comparative illustration of murine and human exhausted monocytes

We then analyzed the monocyte gene expression profiles over time following the onset of murine sepsis. As shown in Fig. 3, we observed that at day 3 post sepsis, infiltrating monocytes within the heart underwent robust exhaustion reflected in pathogenic inflammation (elevated *Cd38, Bst1, ItgaM*) and immune suppression (reduced *Cd86*, and elevated *Cd274*), as well as reduced differentiation and enhanced proliferative potential (elevated *Stmn1*). Many of these key features lasted throughout the 21-day period. Mechanistically, we observed similar activation of *C/ebpβ*, and reduction of *C/ebpα* as well as *Irf5*. Collectively, our systems analyses reveal highly conserved exhaustion profiles of monocytes from human septic patients, murine experimental sepsis, as well as in vitro exhausted monocytes (Fig. 3C). Similar profiles of exhaustion can also be seen in the cycling monocytes (Fig. 3).

### COVID-induced monocyte alterations share key features of monocyte exhaustion seen in sepsis

There are rising interests with regard to COVID-related immune alterations, which may share some similarity with human sepsis. We, therefore, further examined whether the key exhaustion features may similarly exist in monocytes harvested from COVID patients.

**Fig. 4:**
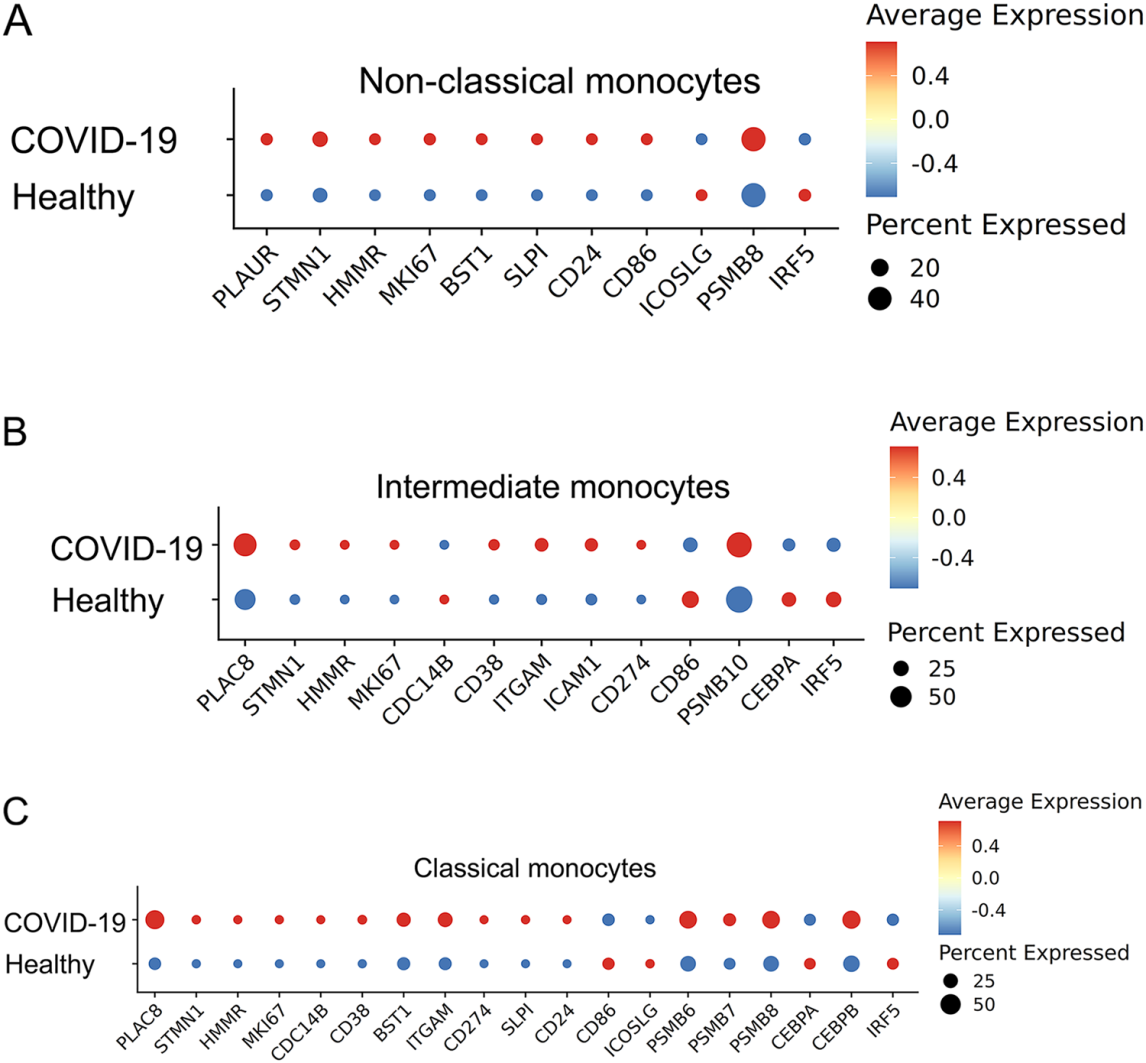
Capturing of key exhaustion maker genes from each subset of monocytes from COVID-19 patients. **A** Dot plot analyses capturing selected genes comparing the non-classical subset of monocytes from healthy or COVID-19 patient blood. **B** Dot plot analyses capturing selected genes comparing the intermediate subset of monocytes from healthy or COVID-19 patient blood. **C** Dot plot analyses capturing selected genes comparing the classical subset of monocytes from healthy or COVID-19 patient blood

As reported through the scRNAseq data, COVID patients similarly experience a reduction of the non-classical population, and an expansion of the classical monocyte population. We, therefore, specifically compared the classical monocytes among healthy and COVID patients. As shown in Fig. 4, we observed similar profiles of enhanced proliferative potential (elevated *STMN1* and *MKI67*), pathogenic inflammation (elevated *CD38, BST1, ITGAM*), and immune suppression (elevated *PD-L1, SLPI, CD24* and reduced *ICOSL*). Mechanistically, we observed elevated proteasome components and increased *C/EBPβ* in COVID patient monocytes. Our data validate that key signatures of monocyte exhaustion are conserved in patients with systemic inflammation caused by COVID-19 infection.

### Auto-immune-monocyte alterations have overlapping yet distinct characteristics as compared to sepsis monocytes

We then turned our attention to chronic low-grade inflammatory diseases, and examined whether monocytes from humans with chronic diseases may have distinct alteration features of monocytes. This is based on our data that monocytes with prolonged challenges with low-grade inflammatory signals bifurcate into a distinct low-grade inflammatory state [3], reflected in pathogenic inflammation and immune-enhancing characteristics.

To test this, we examined recently published scRNAseq data collected from human patients with Vogt–Koyanagi–Harada (VKH) disease, which is a systemic autoimmune disorder characterized by a dysregulated immune response resulting from altered monocyte phenotype and function [8]. Patients with VKH disease exhibit hyperactivity of monocytes and elevated secretion of pro-inflammatory cytokines, which are implicated in the observed inflammation in affected tissues. While the exact cause of VKH disease remains unknown, it is believed to result from a combination of genetic and environmental factors.

**Fig. 5:**
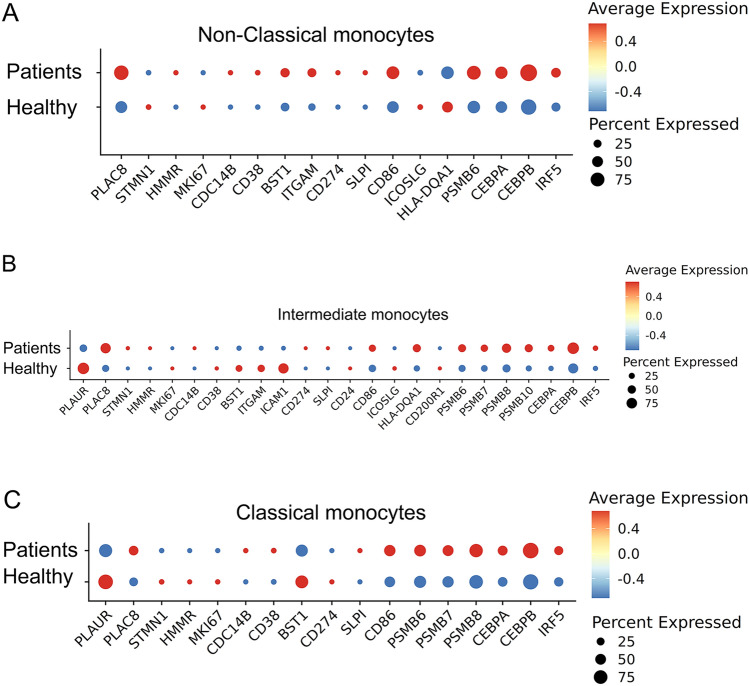
Capturing of key exhaustion maker genes from each subset of monocytes from VKH patients. **A** Dot plot analyses capturing selected genes comparing the non-classical subset of monocytes from healthy or VKH patient blood. **B** Dot plot analyses capturing selected genes comparing the intermediate subset of monocytes from healthy or VKH patient blood. **C** Dot plot analyses capturing selected genes comparing the classical subset of monocytes from healthy or VKH patient blood

We clustered scRNAseq data into three clusters based on CD14 and CD16 expression levels into the non-classical, intermediate, and classical subsets. We then examined representative genes in the categories of pathogenic inflammation, proliferative potential and immune modulation (Fig. 5). In common with the septic patient monocytes, VHK patient monocytes also have the elevated pathogenic inflammation feature with increased expression of *CD38, BST1, ITGAM*. However, in contrast to septic monocytes, we observed that VKH patient monocytes do not exhibit consensus feature of proliferation, suggesting the emergency hematopoiesis and reduced differentiation is not a striking feature of this disease. Furthermore, unlike the septic monocytes, VKH patient monocytes express elevated immune-enhancing genes, such as *CD86*, suggesting the development of the low-grade immune-enhancing phenotype in these patients. Mechanistically, these patient monocytes exhibit an overall elevation of *C/EBPα, C/EBPβ* and *IRF5*. Our analyses reveal that chronic auto-immune types of diseases exhibit distinct features of monocyte reprogramming, with enhanced pathogenic inflammation and immune enhancing characteristics, resembling low-grade inflammatory monocytes we identified in the murine system [3].

**Fig. 6:**
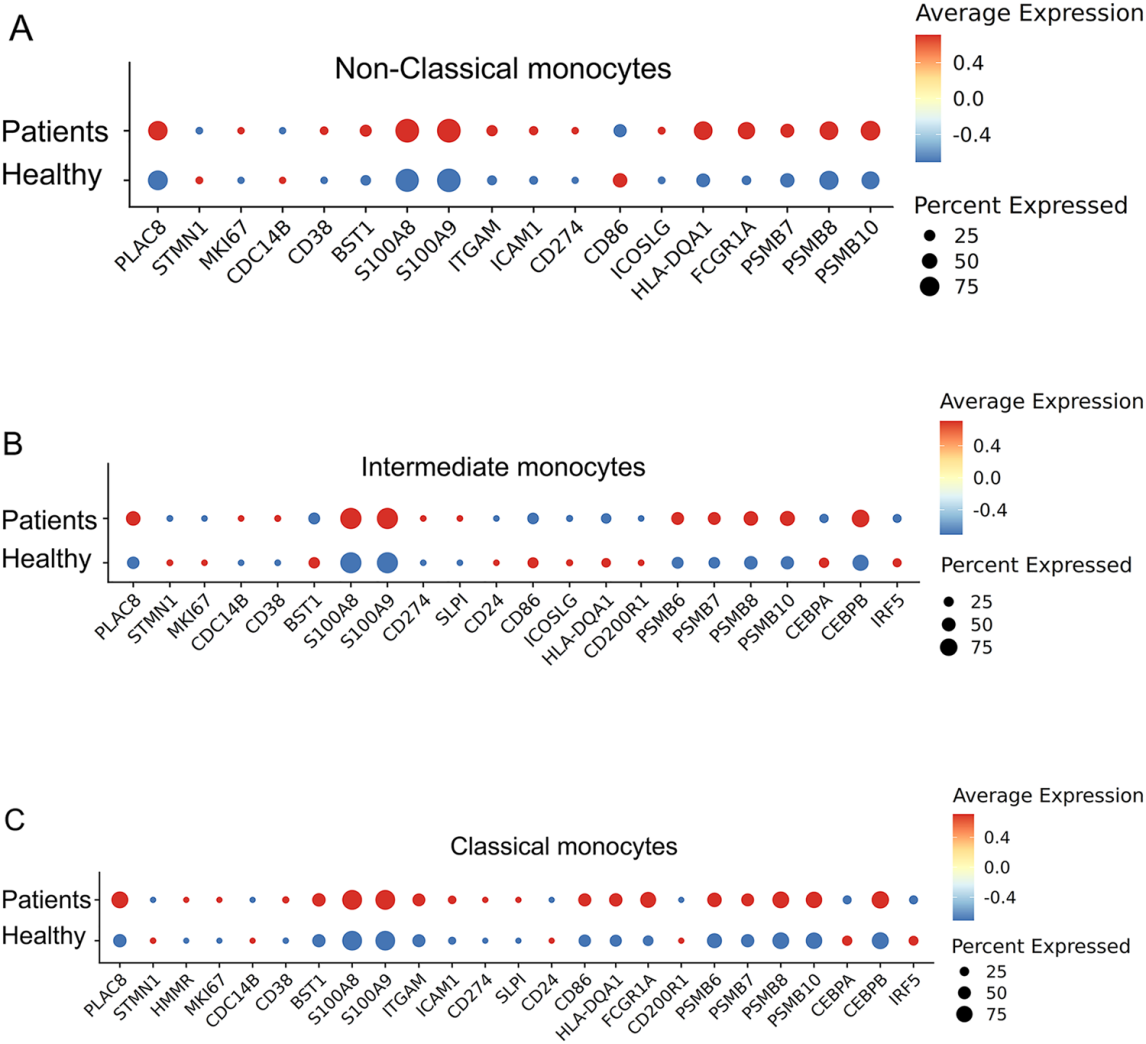
Capturing of key exhaustion maker genes from each subset of monocytes from BD patients. **A** Dot plot analyses capturing selected genes comparing the non-classical subset of monocytes from healthy or BD patient blood. **B** Dot plot analyses capturing selected genes comparing the intermediate subset of monocytes from healthy or BD patient blood. **C** Dot plot analyses capturing selected genes comparing the classical subset of monocytes from healthy or BD patient blood

We further examined another form of human auto-immune disease [10]. Behcet’s disease (BD) is a more severe chronic systemic inflammatory disorder that often presents with recurrent oral/genital ulceration and skin lesions, and in severe cases, can result in multi-organ malfunctions leading to significant morbidity and mortality. Based on recently published scRNAseq data of monocytes collected from BD patients, we similarly grouped BD samples into non-classical; intermediate; and classical clusters, and examined key signatures we identified in this manuscript (Fig. 6). Similar to VHK patients and distinct from septic or COVID-19 patients, the non-classical and intermediate BD patient monocytes do not have a proliferative signature. The pathogenic inflammatory feature, however, is evident from BD patient monocytes with elevated levels of *CD38, BST1, ITGAM*. In contrast to the VHK patients, the intermediate and classical monocytes from BD patients showed higher expression of pathogenic inflammatory genes, as well as reduced immune-enhancing genes, similar to septic monocytes. The “hybrid nature” of semi-exhausted monocytes from the BD patients may correlate with the severe multi-organ dysfunctions.

## Discussion

Our comparative analyses reveal several key principles of monocyte dynamics conserved from human and murine systems. First, prolonged and overwhelming challenges as seen in sepsis initiate and sustain an exhaustion phenotype characterized by reduced differentiation; pathogenic inflammation and immune suppression, which can be recapitulated with prolonged high dose LPS treatment. Second, our pseudo-time analyses suggest that monocytes first halt differentiation and gain proliferative characteristics upon the initial challenge, before bifurcating into either the exhausted state when faced with severe challenges or the low-grade immune-enhancing state when faced with mild challenges. Third, our study reveals conserved molecular circuitry potentially responsible for controlling the memory dynamics in both murine and human monocytes. At the translational level, our analyses demonstrate that blood monocytes from septic and severe COVID patients share overlapping features of exhaustion. In contrast, monocytes from chronic autoimmune patients share distinct features of low-grade inflammation.

Our systems study complements and advances the existing notion regarding the functional characteristics of exhausted monocytes observed in both murine and human sepsis. Previous studies reported the emergence of pre-mature myeloid cells during sepsis, with reduced differentiation and immune suppressive functions, commonly referred as myeloid derived suppressor cells (MDSC) [41]. However, since MDSCs represent a mixture of functionally defined suppressor cells, it has been challenging to clearly illustrate precise cell subsets and underlying mechanisms based on these studies. Recent advances in single cell sequencing analyses ushered in a new era amassing enormous amount of gene expression data from human patients as well as experimental animal. Although these studies are extremely information-rich, they are less informative without a proper integration with conventional hypothesis-based studies. There is an urgent need to make a clear and better sense of these large data sets and define key causal nodes closely related to the pathogenesis of sepsis as well as other inflammatory diseases. To this regard, we examined key targets genes with relevant conventional pathogenesis perspectives from the large data sets representing human sepsis as well as animal experimental sepsis. These target genes were selectively mined from the large data sets to represent key principles of monocyte exhaustion, namely, enhanced proliferation; pathogenic inflammation and immune suppression, known to play causal roles during sepsis pathogenesis. We demonstrate that these target genes can be conservatively captured from previously published scRNAseq data sets collected from human sepsis patients. Comparatively, we demonstrate that these key gene expression features can also be captured from the scRNAseq data sets from the murine model of sepsis. Our comparative analyses posit that these key gene signatures should be further examined through the conventional hypothesis-driven experimental approaches in the future, with well-defined healthy and control patients, to potentially serve as diagnostic markers for sepsis.

Although previous approaches eluded to the proliferative and pathogenic nature of septic myeloid cells, underlying mechanisms are still poorly defined. Taking advantage of single cell sequencing data, we were able to perform pseudo-time trajectory analyses to pinpoint the temporal ontogeny of septic monocytes. Our analyses reveal an intriguing clue indicating that septic monocytes initially undergo halted differentiation and re-gain proliferative potential, correlating with previously identified “emergency hematopoiesis” during sepsis [42]. Following the initial de-differentiation, septic monocytes subsequently gain the next set of exhaustion features including pathogenic inflammation and immune suppression. Based on our analyses, future experimental validation is warranted to test whether cell proliferation inhibitor may block subsequent monocyte exhaustion, and whether such strategy can be used for treating sepsis.

Our data further reveal that the initial de-differentiation process is a common gateway for monocytes facing endotoxin challenges with varying signal strength. Our scVelo analyses reveal that monocytes with subclinical super-low dose LPS challenge similarly go through the initial de-differentiation step. This is consistent with previous genetic and functional studies reporting that enhanced clonal hematopoiesis is closely associated with exacerbated atherosclerosis [43–45]. In contrast to the higher dose LPS challenge, super-low dose LPS nudges monocytes to the low-grade inflammatory states, sequentially from the initial de-differentiated state to immune-enhancing, pro-growth state and the late mature state with inflammatory and adhesive properties. The signatures of chronic inflammatory monocyte states can be partially seen in monocytes from chronic human diseases such as atherosclerosis and various forms of auto-immune diseases. Our comparative analysis reconciles previous studies that reveal persistent low-grade “metabolic endotoxemia” in circulation (~ 1–100 pg/ml) as a closely related risk factor for chronic metabolic diseases, such as diabetes and atherosclerosis [46–49]. In contrast, higher doses of circulating endotoxin (~ ng/ml) are present in human septic patients [50, 51].

Taken together, our systems analysis not only reveals conserved features of monocyte exhaustion, but also provide important insights into the molecular mechanisms underlying monocyte exhaustion in sepsis, highlighting alterations in gene expression profiles related to cell differentiation/proliferation, pathogenic inflammation, and immune suppression, as well as the involvement of specific signaling components and transcription factors. However, our current work is limited in scope by attempting to reconcile emerging scRNAseq studies amassing large amount of data sets with hypothesis-driven studies on selected targets utilizing well-controlled human or experimental animals. Although our comparative studies comparing in vitro and in vivo scRNAseq data suggest some intriguing principles and reveal promising target genes likely representing key signatures of monocyte exhaustion or low-grade inflammation, future independent in vivo studies with well-controlled experimental groups should be pursued to provide causative validation based on this initial comparative analyses. Further understanding of these mechanisms through future studies may potentially lead to the development of novel therapeutic strategies for sepsis and other inflammatory conditions involving monocyte dysfunction.

## Data Availability

This study utilized data sets publicly available as indicated in the “Materials and methods” section.
